# A novel low shrinkage dimethacrylate monomer as an alternative to BisGMA for adhesive and resin-based composite applications

**DOI:** 10.1590/1807-3107bor-2024.vol38.0097

**Published:** 2024-09-30

**Authors:** Fernanda Sandes de LUCENA, Matthew LOGAN, Steven LEWIS, Neil DEATHERAGE, Adilson Yoshio FURUSE, Carmem Silvia PFEIFER

**Affiliations:** (a) Universidade de São Paulo – USP, Bauru School of Dentistry, Department of Operative Dentistry, Endodontics and Dental Materials, Bauru, SP, Brazil.; (b) Oregon Health & Science University, School of Dentistry, Department of Restorative Dentistry, Portland, OR, EUA.

**Keywords:** Composites, Dental Cements, Dental Materials, Rheology

## Abstract

The aim of this study was to develop a mixture of dimethacrylate isomers (PG6EMA) as a potential monomer for dental adhesives and composites. PG6EMA was synthesized de novo and characterized in the presence of ethanol (3%, 6% or 9%). BisGMA/TEGDMA (BTEG, 50/50 wt.%) was used as the resin control. Composites were formulated with 60 wt.% of either PG6EMA or BisGMA (40 wt.% TEGDMA and 70 wt.% filler). DMPA (0.2 wt.%) and DPI-PF6 (0.4 wt.%) were added as photoinitiators, irradiated with a mercury arc lamp (320–500 nm, 500 mW/cm^2^; Acticure). All materials were tested for polymerization kinetics (near-infrared), viscosity (η) and storage modulus (G’, oscillatory rheometry). The composites were further characterized for water sorption/solubility, wet/dry flexural strength/modulus and polymerization stress. Data were analyzed with one-way ANOVA/Tukey’s test (α = 0.05). The PG6EMA resins showed lower rates of polymerization compared with BTEG (p = 0.001) but high degrees of conversion (p = 0.002). Solvent concentration did not affect RP_MAX_ but the 6% and 9% mixtures showed higher final DC, likely due to reduced viscosity. PG6EMA had much higher viscosity than BTEG (p <0.001) and lower G’ (p = 0.003). Composites modified with PG6EMA have slower polymerization rates (p = 0.001) but higher final DC (p = 0.04) than the control. PG6EMA/TEGDMA showed lower dry/wet flexural strength and comparable dry modulus. The PG6EMA/TEGDMA composite showed a 18.4% polymerization stress reduction compared to the BTEG composite. Both base monomers had similar WS/SL and G’. Within its limitations, this study demonstrated that the newly synthesized PG6EMA was a viable alternative to BisGMA in dental composites.

## Introduction

Resin-based materials play an important role in dentistry and can be used for a large range of clinical applications, such as dental adhesives, resin composites, cavity liners, pit and fissures sealants, core buildups and the cementation of orthodontic devices and indirect restorations.^1^ The typical formulation of dental composites consists of four key components: an organic matrix (mainly dimethacrylate monomers); inorganic reinforcing fillers; coupling agents; and a photoinitiator system. Usually, a combination of monomers is employed for these formulations and this mixture can influence viscosity, reactivity, mechanical properties, water sorption and solubility and polymerization shrinkage.^2^ For adhesives, the same composition can be used, requiring the addition of a solvent (ethanol, water or acetone) to decrease the viscosity and improve the wettability and diffusion of monomers into the collagen network.^3^

Among the available monomers for the polymeric organic matrix, the most commonly used, since the 1960s, is bisphenol A-glycidyl methacrylate (BisGMA).^4^ This monomer imparts good mechanical properties, mainly due to the presence of hydroxyl groups that establish hydrogen bonds in the alkyl chain (which also significantly increase viscosity), and π-π interactions about the aromatic rings (molecular weight of 512.6 g/mol and η of 500–1,200 Pa.s).^5^ The high viscosity imparts the addition of adequate amount of inorganic filler particles to the composite, which necessitates the use of a low viscosity aliphatic crosslinker monomer, such as triethylene glycol dimethacrylate (TEGDMA, molecular weight of 286 g/mol and η of 0.05 Pa.s) to work as diluent and reduce BisGMA viscosity, with the additional effects of improving handling properties^5^ and increasing the mobility of the reaction media, ultimately resulting in higher conversion.^5,6^ Notwithstanding, TEGDMA increases network heterogeneity due to primary cyclization^5^ and also increases polymerization shrinkage, potentially causing microgap formation, which in turn may lead to bacteria infiltration, secondary caries and restorative treatment failure.^7^ It is important to point out; however, the mechanism for secondary caries formation is complex, so, other than gap formation factors related to the patient (dietary and hygiene habits, timeline of specific bacteria colonization and resulting microflora) also need to be considered.^8^ Specifically, for use in adhesives, where the mechanical demands are less stringent than in composites, increased conversion and decreased leachability of potential cytotoxic components that can reach the pulp are additional desirable characteristics. It is well-documented that BisGMA/TEGDMA systems also display adverse effects on cellular activities, because residual monomers can leach out from restorative materials due to unreacted methacrylic groups during polymerization reaction affecting the metabolism and growth of pulp tissue and cells.^9^

Several randomized controlled clinical trials and follow up studies have demonstrated the increase in longevity in dental composite restorations since their introduction on the clinician’s armamentarium over 60 years ago.^10,11^ However, despite these improvements, several cohort studies from practice-based research networks still demonstrate an average longevity of 12 years, which is much shorter than amalgams.^12,13^ To overcome drawbacks related to the use of these materials in dentistry, any new monomers need to perform at least similarly to BisGMA in terms of mechanical properties, with improvements needed in terms of conversion and cytotoxicity. Several new monomers that can reduce or replace BisGMA from their composition are being explored.^14-16^ New strategies include the use of high molecular weight monomers^17^ or nanogel prepolymers^18^ to reduce shrinkage, thiourethane oligomers^19^ or the use of thiol-ene systems,^20^ in which stress reduction is achieved by delaying polymer vitrification but increasing conversion. More recently, other strategies focusing on novel chemistries have been investigated, such as use of acrylamides,^21^ vinyl ethers,^22^ copper-catalyzed alkyne-azides^23^ and so on. In all these cases, the objective was to reduce susceptibility to hydrolysis in the oral environment, increase restoration longevity and reduce cytotoxicity concerns. Any of these monomers can conceivably be applied in the composition of solvated adhesives or highly filled composites.

Therefore, the objective of the present study was to synthesize alternative monomers and characterize BisGMA-free compositions that could be potentially used in copolymer formulations for dental adhesives and composites. PG6EMA was a high molecular weight monomer, synthesized de novo as a combination of isomers. The molecular structure was designed to increase the stability of the methacrylate bond with a piperidine ring. In this study, PG6EMA was tested as the sole monomer system (mixture of isomers) associated with serial amounts of ethanol as the solvent or as a copolymer with TEGDMA for potential use in composites. The materials were tested for polymerization kinetics, mechanical properties, rheological behavior and water sorption/solubility and compared to materials in which the base monomer was BisGMA. The hypotheses of this study were that this new mixture of isomers would exhibit lower polymerization stress and comparable mechanical properties when compared to a BisGMA-based resin and composite to be used in dental adhesives and composite formulations. BisGMA/TEGDMA-based materials were used as controls for PG6EMA.

## Methods

In this study, materials containing a new mixture of isomers (PG6DMA) were tested for their adhesive and composite applications. The base monomers for the control groups consisted of BisGMA/TEGDMA for either type of material. Additionally, for the composites, a microhybrid mixture of fillers was utilized at a loading level similar to commercially available composites. Although DMPA was used as a single component, α-cleavage type initiator for simplicity, a conventional camphorquinone/amine system (CQ/amine) was also compatible with the methacrylate chemistry of the isomers. The formulations are detailed below. It should also be noted that even though a mercury arc lamp was used in this study for practical reasons (continuous irradiation for the duration of the kinetics and bar fabrication procedures), any dental light curing unit could be used instead to cure a CQ/amine-based material.

### Materials formulation

#### PG6EMA synthesis

**Figure 1:**
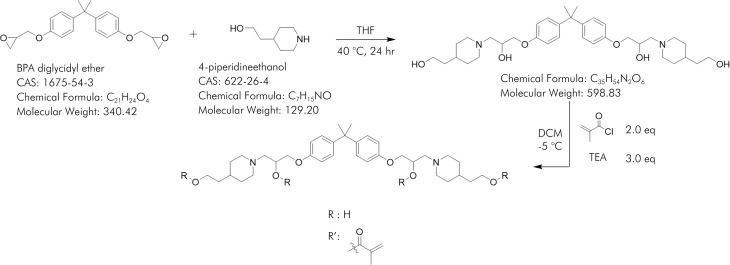
PG6EMA synthetic scheme. The resulting product is mixture of dimethacrylate isomers.

**Figure 2:**
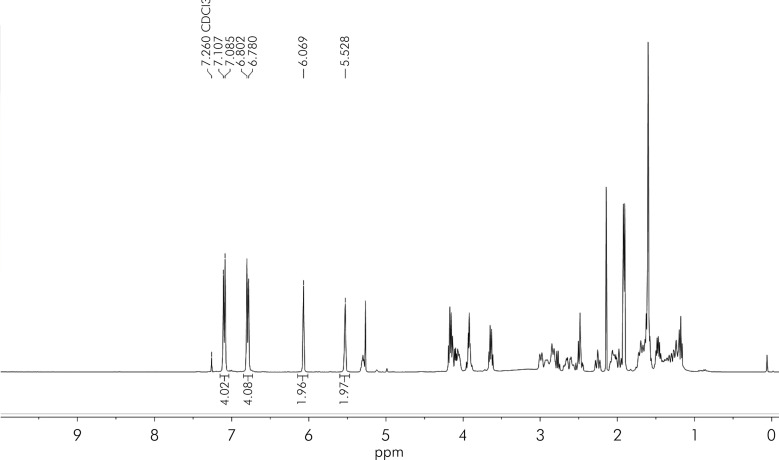
1H NMR (CDCl3) of PG6EMA mixture of isomers. The aryl proton peaks (7.1 and 6.8 ppm) and vinyl proton peaks (6.1 and 5.5 ppm) integration areas give a ratio of 2:1, indicating that successful synthesis of a dimethacylate.

The reaction scheme is shown in Figure 1. A solution of Bisphenol A diglycidyl ether (5.0 g, 14.7 mmol) and 4-piperidineethanol (4.1 g, 29.4 mmol) in 50 mL of tetrahydrofuran was heated to reflux for 16 h. After cooling to room temperature, the solution was diluted with 100 mL of ethyl acetate. The organic residue was washed three times with 0.1 M NaOH (30 mL), once with brine (30 mL) and dried over Na_2_SO_4_. The solution was concentrated at 25°C, resulting in slight yellow viscous oil. Without further purification, the oil was redissolved in dichloromethane (80 mL) and triethylamine (6.1 mL, 44.1 mmol) was added. The solution was cooled to -5°C, followed by the slow addition of a solution of methacryloyl chloride (3.1 g, 29.4 mmol) in dichloromethane (20 mL). The solution was allowed to warm to room temperature and was stirred for 16 h. After filtration through celite, the filtrates were washed with saturated NaHCO_3_ (20 mL), brine (20 mL) and dried over Na_2_SO_4_. The solution was concentrated at 25°C resulting in a viscous, slightly orange oil. An NMR spectrum (^1^H, CDCl_3_, Figure 2) was obtained and the ratio of aryl protons to vinyl protons was used to confirm that a dimethacrylate had been produced. This mixture of PG6EMA isomers was used without further purification.

#### Resin formulation

PG6EMA presented as a high viscosity oil, similar to BisGMA. To test it as a homopolymer (mixture of isomers), absolute ethanol (EtOH) was added to the unfilled materials at 3, 6 and 9 wt.%. The EtOH concentrations were chosen to mimic solvent levels present in the clinical application of dental adhesives, where even after prolonged evaporation, 4–9% residual solvent still remains in comonomer-ethanol systems (as previously determined in vitro).^24^ BisGMA/TEGDMA mixtures (50/50 wt.%), both from Esstech (Essington, USA), were used as the controls with the same solvent levels. The photoinitiator system consisted of 0.2 wt.% of the alpha-cleavage type, single component 2,2-dimethoxy-2-phenyl acetophenone (DMPA) and 0.4 wt.% DPI-PF_6_ (diphenyl iodonium hexafluorophosphate). Furthermore, 0.1 wt.% of butylated hydroxytoluene was incorporated as an inhibitor. The materials were tested immediately after solvent incorporation.

#### Composites

Composites were formulated using either PG6EMA or BisGMA combined with 50 wt.% TEGDMA. Filler particles were added at 70 wt.% (of which 5% were OX-50, 50 nm and 95% were 0.7 µm barium glass, ESSTECH). The initiator system was identical to the one used for the resin formulations described above. The mixture was made in a mechanical mixer (SpeedMixer DAC 150 FVZ; Flacktek, Landrum, SC, USA) at 1,600 rpm for 60 s.

## Polymerization kinetics and degree of conversion

Disk samples (n = 3) were prepared in 0.8 mm thickness × 10 mm diameter silicon molds between two glass slides. The degree of conversion (DC) was accessed using near-infrared (near-IR) spectroscopy (Nicolet 6700; Thermo Fisher Scientific, Waltham, MA, USA) in real time during photopolymerization. The methacrylate double-bond absorption at 6165 cm^1^ was recorded during 300 s of irradiation at 500 mW/cm^2^ (Acticure 4000, 320–500 nm) and used to calculate the DC.^25^ The polymerization rate was calculated as the first derivative of the DC versus time curve.

## Mechanical and rheological properties

### Flexural strength and modulus of elasticity

The flexural strength (FS) and modulus of elasticity were assessed using a three point bending test. Twelve bars with 2.0 mm width × 2.0 mm thickness × 25.0 mm length per group were made using a silicone mold sandwiched between glass slides. The samples were light cured using the same conditions as in the polymerization kinetics, i.e., 120 s on each side. The bars (n = 6) were stored for 48 h in Millipore water or in dry conditions and, after this period, the test was carried out in a universal test machine at a crosshead speed of 0.5 mm/min until fracture. The FS was calculated using the following equation:^26^


$$FS=\frac{3FL}{2wh^{2}}$$

Where:

*FS*: flexural strength (MPa)

*F*: load at fracture (N)

*L*: span length (mm)

*w:* sample width (mm)

*h*: sample thickness (mm)

The modulus of elasticity (GPa) was calculated according to the below equation:


$$E=\frac{FL^{3}}{4bh^{3}d}$$

Where:

*E*: modulus of elasticity (GPa)

*F:* load at some point of the linear region of the stress-stain curve (N)

*L:* distance between the supports of the sample (25 mm)

*b:* sample width (2 mm)

*h*: sample thickness (mm)

*d*: slack compensated deflection at load F

## Storage modulus and viscosity

Approximately 0.5 g of each material (n = 3) was placed between 8 mm diameter parallel acrylic plates attached to a rheometer (DHR-1; TA Instruments, New Castle, DE, USA). The storage modulus (G’) was determined with a 10 Hz frequency and 0.1% strain during polymerization. Given the limitations with the data acquisition speed for the rheometer (one data point per s), this test was carried out using the same light source at lower irradiance (250 mW/cm^2^) for 600 s. The viscosity was measured at 1 Hz with a gap of 300 µm (n = 6).

## Water sorption and solubility

This test was performed according to ISO4049 ^26^. The composite disks used for polymerization kinetics (n = 3, 10 mm diameter × 0.8 mm thickness) were also used for water sorption and solubility. The disks were weighted on a precision scale to obtain the initial mass (m1) before storing the samples in a glass vial with 5 mL of Millipore water for 7 d. After this time, the excess water was removed with absorbent paper and the mass (m2) was recorded. The samples were then placed on a vacuum desiccator to dry until a constant mass (m3) was obtained. The water sorption (WS) and solubility (SL) were calculated following the equations:


$$WS=\frac{m2-m1}{V_{o}}\;SL=\frac{m2-m3}{V_{o}}$$

Where:

WS: water sorption

SL: solubility

m1: sample mass after the first drying (mg)

*m*2: sample mass after immersion in water (mg)

*m*3: sample mass after the second after the second drying (mg)

Vo: sample volume (mm^3^)

## Polymerization stress

The polymerization stress was assessed using a cantilever beam apparatus (Bioman)^27^ and recorded in real time. For this test, a 5 mm diameter × 0.5 mm tall steel rod load cell was used and the rod bottom needed to be previously treated with a metal primer (Z-prime plus; Bisco, Schaumburg, USA). To hold the composite sample, a 3-mm-thick silica glass plate was used and treated with a silane agent (RelyX ceramic primer; 3M ESPE, St. Paul, , USA). The resin composite samples (n = 3) were inserted in the 0.5 mm gap between the metal rod and the silica glass plate and shaped into a disk. The samples were photocured at the same conditions used for polymerization kinetics, i.e., for 300 s.

## Statistical analysis

Data were analyzed for normality (Anderson-Darling) and homoscedasticity (Bartlett and Levene). Statistical analysis was carried out using the t-test or one-way ANOVA, followed by Tukey’s post-hoc test for multiple comparisons. An overall level of significance of 5% was adopted for all tests.

## Results

### Unfilled materials

**Figure 3:**
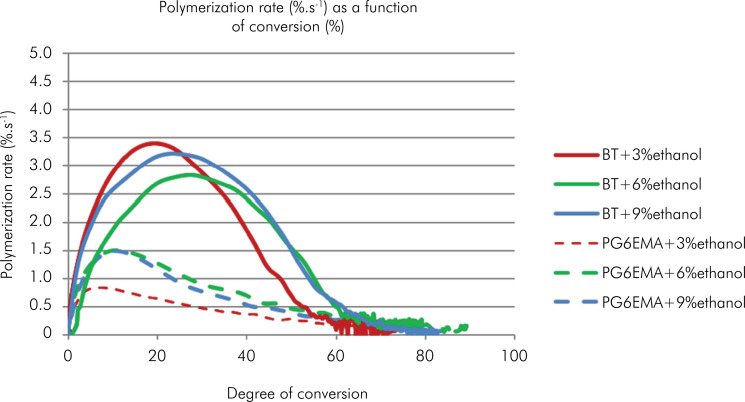
Degree of conversion as a function of the rate of polymerization for unfilled BT formulation with 3, 6 and 9 EtOH% and PG6EMA with the same EtOH%. Vinyl pick followed for 300 seconds with exposure irradiance of 600 mW/cm2.

The polymerization kinetics curves for unfilled materials (BT and PG6EMA) with serial amounts of EtOH (3, 6 and 9 wt.%) are shown in Figure 3 and the average/standard deviations are presented in Table 1. The maximum rate of polymerization (RP_max_) was higher for all BT groups, regardless of the EtOH concentration. The DC at RP_max_ was used as a proxy for vitrification onset. The highest values were found for BT containing 6% and 9% EtOH (27.8±1.4% and 26.1±1.4%, respectively). The lowest results were for PG6EMA with 3% and 6% EtOH (8.4±1.7% and 7.9±1.3%, respectively). The final DC was higher for PG6EMA groups, especially for the 6% and 9% formulations, reaching final conversions of 91.1±2.5% and 90.7±0.5%, respectively.

**Table 1:** Mean and standard deviation RPMAX (% s-1), DC at RPMAX (%) and final DC (%), viscosity (η) and storage moduli (G’) for all homopolymer and BTEG control groups.

Variable	RP_MAX_ (%.s^-1^)	DC at RP_MAX_ (%)	Final DC (%)	η (Pa.s)	G’ (MPa)
BT:3%	3.4 (0.3)^a^	18.6 (0.8)^b^	73.5 (3.2)^b^	0.04 (0.00)^c^	130.9 (15.2)^a^
BT:6%	3.4 (0.3)^a^	27.8 (1.4)^a^	79.8 (2.2)^ab^	0.03 (0.00)^c^	105.8 (18.4)^a^
BT:9%	3.3 (0.2)^a^	26.1 (1.4)^a^	84.4 (1.7)^ab^	0.04 (0.00)^c^	112.0 (16.2)^a^
PG6EMA:3%	1.0 (0.3)^b^	8.4 (1.7)^c^	80.7 (5.2)^a^	8.06 (1.56)^a^	60.3 (15.7)^b^
PG6EMA:6%	1.5 (0.4)^b^	7.9 (1.3)^c^	91.1 (2.5)^a^	3.21 (1.52)^b^	26.0 (19.5)^b^
PG6EMA:9%	1.7 (0.2)^b^	12.4 (0.0)^bc^	90.7 (0.5)^a^	0.16 (0.03)^c^	30.7 (6.5)^b^
p-value	0.001	<0.001	0.002	<0.000	0.003

Same lowercase letters within the same column denote statistically similar values (α = 0.05).

The results for viscosity and G’ are displayed in Table 1 . It should be noted that the viscosity of neat PG6EMA was similar to BisGMA (~1,200 Pa.s). With the addition of solvent, which is relevant to the adhesive application, all BT groups presented similar viscosity results regardless of EtOH%. For PG6EMA, the increasing EtOH% significantly decreased the viscosity, ranging from 8.06 ± 1.56 (3% EtOH) to 0.16 ± 0.03 (9% EtOH) Pa.s. A total of 9% EtOH PG6EMA had comparable viscosity results to all BT groups. G’ was not affected by the addition of ethanol for both the BT and PG6EMA groups, but all BT groups showed higher G’ compared to the PG6EMA groups. G’ ranged from 26±19.5 to 60.3 ± 15.7 MPa for BT 3% and 9% EtOH, respectively, while the PG6EMA groups ranged from 26±19.5 to 60.3 ± 15.7 MPa (6% and 3% EtOH, respectively).

### Filled materials (composites)

**Figure 4:**
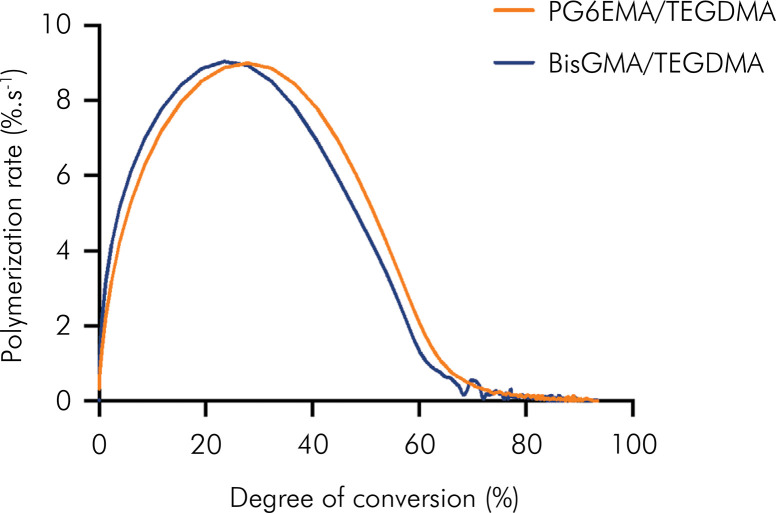
Degree of conversion as a function of the rate of polymerization for control and PG6EMA composites. Vinyl pick followed for 300 seconds with exposure irradiance of 600 mW/cm2.

The average for the three polymerization kinetics curves is displayed in Figure 4 and averages/standard deviations are shown in Table 2 . The same trend found for the unfilled materials was observed, i.e., the PG6EMA-based composite showed lower RP_max_ (9.1 ± 0.1%.s^-1^) and DC at RP_max_ (27.8 ± 0.8%) but higher DC (93.4 ± 1.6%) compared to the BisGMA:TEGDMA control. As for polymerization stress, PG6EMA copolymerized with the TEGDMA composite showed a significantly lower value (6.2 ± 0.1 MPa) compared to the BisGMA/TEGDMA composite (7.5 ± 0.7), i.e., which was an 18.4% stress reduction ( Table 2 ).

**Table 2:** Mean and standard deviation RPMAX (% s-1), DC at RPMAX (%) and final DC (%) and polymerization stress (MPa) for all homopolymer and BTEG control groups.

Variable	RP_MAX_ (%.s^-1^)	DC at RP_MAX_ (%)	Final DC (%)	Polymerization stress (MPa)
PG6EMA/TEGDMA	9.1 (0.1)^b^	27.8 (0.8)^b^	93.4 (1.6)^a^	6.2 (0.1)^b^
BisGMA/TEGDMA	9.7 (0.1)^a^	30.2 (1.0)^a^	87.8 (2.7)^b^	7.5 (0.7)^a^
p-value	0.001	0.03	0.04	0.02

Same lowercase letters within the same column denote statistically similar values (α = 0.05).

The mechanical properties of the composites are shown in Table 3 . PG6EMA/TEGDMA presented lower dry and wet flexural strength (76.5 ± 6.5 and 56.9 ± 12.3 MPa, respectively) compared to BisGMA/TEGDMA (99.9 ± 10.6 and 82.8 ± 10.8 MPa). Both composites showed comparable dry modulus of elasticity (7.4±0.6 GPa for BisGMA/TEGDMA and 6.8 ± 0.3 GPa for PG6EMA/TEGDMA). The wet modulus of elasticity decreased 25% for the PG6EMA composite (5.1 ± 0.7 GPa) but did not change for the BT control composite (7.3 ± 1.2 GPa). G’ was similar for both composites, 151.9 ± 19.7 MPa for PG6EMA/TEGDMA and 156.1 ±1 5.4 MPa for BisGMA/TEGDMA ( Table 3 ). The results for water sorption and solubility are also shown in Table 3 . For WS and SL, there was no significant difference between the composites. In terms of WS, the results were 26.5 ± 2.0 µg/mm^3^for BisGMA/TEGDMA and 33.4 ± 4.8 µg/mm^3^ for PG6EMA/TEGDMA composite. As for SL, the results were 67.4 ± 2.6 and 71.1 ± 1.7 µg/mm^3^, respectively.

**Table 3:** Mean (standard deviation) of flexural strength (FS) and modulus of elasticity (E) after 24 h of dry storage and 7 d of water storage, shear storage moduli (G’), and water sorption (WS) and solubility (SL) results for BISGMA/TEGMA and PG6EMA/TEGDMA composites.

Varuable	FS (MPa)		E (GPa)		G’ (MPa)	WS	SL
	Dry	Wet	Dry	Wet		µg/mm^3^	µg/mm^3^
PG6EMA/TEGDMA	76.5 (6.5)^bA^	56.9 (12.3)^bB^	6.8 (0.3)^aC^	5.1 (0.7)^bD^	151.9 (19.7)^a^	33.4 (4.8)^a^	71.1 (1.7)^a^
BisGMA/TEGDMA	99.9 (10.6)^aA^	82.8 (10.8)^aB^	7.4 (0.6)^aC^	7.3 (1.2)^aC^	156.1 (15.4)^a^	26.5 (2.0)^a^	67.4 (2.6)^a^
p-value	0.001	0.003	0.050	0.011	0.268	0.165	0.055

Same lowercase letters within the same column denote statistically similar values (Tukey’s test, α = 0.05). For comparisons between dry and wet results, similar uppercase letters denote statistically similar values (*t*-test, α = 0.05)

## Discussion

The main purpose of the present study was to design, synthesize and characterize PG6EMA as a potential BisGMA-free substitute and to compare those formulations to typical BisGMA/TEGDMA mixtures.^28^ The screening properties were evaluated with the aim of providing information for designing adhesive and composite materials as part of future studies. In fact, the results showed that this monomer was capable of reducing polymerization stress while increasing conversion without compromising shear modulus.

All solvated PG6EMA groups showed higher DC compared with BisGMA/TEGDMA mixtures, albeit with statistical significance only for 3% BT. The increase in solvent concentration led to similar increases in conversion for both materials, which was a ~10% increase from 3% to 9% EtOH. The higher conversion for PG6EMA is explained in part by its higher molecular weight compared to the BisGMA/TEGDMA mixture, which reduces the vinyl concentration per unit volume of monomer and therefore leads to a reduction in volumetric shrinkage upon polymerization^6^. In addition, the absence of the less reactive TEGDMA in the PG6EMA formulations likely also contributed to higher conversion, in spite of the fact that the DC at RP_MAX_ for PG6EMA was lower when compared to BT materials. High values of DC (70% or above) are desirable for dental adhesives to increase the stability of the formed polymer network and to reduce the potential cytotoxicity caused by unreacted monomers ^29^. The DC at RP_MAX_ was used here as a proxy for the onset of vitrification and it was expected that higher values would translate in higher final conversion, which was not observed here. One potential explanation is that TEGDMA is highly prone to primary cyclization, which contributes to the overall values of conversion, but not network formation because the double bonds that are consumed do not necessarily get incorporated into the resulting polymer.^6^ Therefore, and likely potentiated by the presence of solvents,^6^ the crosslinking density might have been lower in those groups, increasing the potential for conversion prior to vitrification.

Past vitrification, the availability of monomers to react is lower in BTEG compared to PG6EMA, therefore leading to lower conversion overall. The rate of polymerization was lower for PG6EMA and the solvent content did not affect RP_MAX_ for any of the materials (Figure 3 and Table 1). The lower rate of polymerization may be partially explained by the higher initial viscosity of PG6EMA (Table 2),^30^ which caused the environment of the chemical reaction of polymerization to be sterically hindered. In fact, even at 9% EtOH concentration, PG6EMA had higher viscosity than BT at the lowest EtOH concentration tested (3%). This also meant that vitrification was reached at lower conversions, which was indeed observed with the DC at RP_MAX_ results. However, because of the presence of solvent in the system, the polymerization still continued past what would normally be expected. Indeed, the 9% EtOH mixture showed statistically higher DC at RP_MAX_ and both 6% and 9% mixtures showed higher final DC. Overall, the increased EtOH concentration and consequent drop of viscosity also explained the delayed deceleration and ultimately the higher final DC (Figure 3).^31^

Highly filled resin composite applications require materials with relatively high values of mechanical properties and low polymerization stress.^32^ However, in adhesive formulations these characteristics are less important because these materials are not under direct mechanical loading and polymerize with little constraint by cavity walls (low C-factor).^29^ For adhesives, high DC in the presence of solvents and high resistance to degradation are crucial.^33^ The incorporation of solvents is necessary to decrease material viscosity, therefore allowing for better wettability of the water-rich dentin environment, and also to facilitate water displacement and the infiltration of adhesive resin into dentin.^34^ Even though most of the solvent is removed prior to polymerization, some studies estimate 5–13% still remains in the system, especially in those materials containing ethanol, and that percentage also depends on the time of evaporation.^35^

Other factors such as the nature of the monomers in the composition, their vapor pressure, solvent molecular weight and the amount of water in dentin also influence the amount of solvent leftover after evaporation procedures.^34^ Evidently, solvents do not participate in network formation, and they may jeopardize mechanical properties and polymerization kinetics, reducing final degree of conversion and bond strength.^34^ Previous studies demonstrated that the presence of solvent in amounts around 15−20% compromised the mechanical properties of methacrylate-based systems.^36^ One goal of the present study was to evaluate solvent interaction with the newly synthesized PG6EMA homopolymer for adhesive formulations compared to BisGMA/TEGDMA. For that purpose, ethanol was the solvent of choice, added to the unfilled materials in serial amounts of 3, 6 and 9 wt.%, within the range reported in the literature.

The use of solvents in this study was done for two reasons: the first, was of a practical nature, to allow for handling of the highly viscous PG6EMA and to evaluate its properties as a homopolymer. For perspective, PG6EMA has viscosity similar to pure BisGMA. The second reason relates to the potential utilization of this monomer as the base of a dental adhesive. Because BisGMA is not normally used as a homopolymer in those situations, the control group for PG6EMA was a mixture of BisGMA/TEGDMA at 50/50 wt.%. When the shear storage modulus of the different solvated formulations was compared, the BT groups presented results that were two- to three-fold higher than PG6EMA. This may be explained by the much shorter crosslinks formed with the BT groups, compared with the longer and more flexible PG6EMA, which could have contributed to a stiffer network. As mentioned, this might not be a limitation in terms of adhesive applications, because previous studies have demonstrated that even with much lower properties after water storage, certain monomers are still able to produce high values of dentin bond strength^21^ Future studies for this application will include evaluation of degradation by hydrolysis and enzymatic attack, and bonded interface stability.

Composite materials were also evaluated using either BisGMA or PG6EMA as base monomers in combination with TEGDMA as a diluent and added inorganic fillers. For the highly filled materials, the polymerization kinetics profile was very similar among the groups, with statistically similar rates of polymerization (autoacceleration and deceleration) and conversion at maximum rate. However, the conversion for PG6EMA-based materials was statistically higher, for the same reasons already explored in the solvated systems. The same samples obtained after polymerization kinetics were incubated in water to evaluate the water sorption (WS) and solubility (SL), and no difference was found between the groups. The values for both WS and SL were very low, likely due to the high final degree of conversion obtained for both groups. The dry elastic modulus (E) and the shear storage modulus (G’) were comparable for PG6EMA composite and BT control, which meant the effects of PG6EMA on polymerization stress were not attributable to decreased material stiffness. However, flexural strength (FS) was higher for BisGMA/TEGDMA system for both dry and wet bars, and while the modulus for BT materials remained stable after water storage, the PG6EMA materials having a 25% drop in the elastic modulus was observed for PG6EMA composite after 7 d of water storage. The drop of properties in aqueous environment was a limitation of this material for composite applications, and warrants further investigation of potential co-monomers, in addition to polymeric additives with capability of reducing network swelling.^37^ However, as already mentioned, the solubility of this material was similar to the Bis-GMA-based materials, meaning that the reduction in properties could not be attributed to material degradation. Instead, this indicated that, again, the length scale of the crosslinks might be playing a role here. In fact, studies investigating the free-volume of BisGMA-TEGDMA networks have demonstrated that both the final conversion and the flexibility of crosslinks influenced the amount of water and organic solvent uptake.^38^

The polymerization stress of PG6EMA/TEGDMA composite was 18.4% lower compared to the BisGMA/TEGDMA control. This was true in spite of the increase in conversion (about 6% higher for the PG6EMA material) and similar dry flexural and shear moduli. Because delayed gelation/vitrification (inferred by the similar conversion at maximum rate of polymerization) were neither observed nor expected, and because the dry moduli (shear and flexural) were similar for both materials, the lower polymerization stress might be explained by the high molecular weight of PG6EMA, which reduces its molar shrinkage coefficient.^39^ In addition to lower polymerization stress, this material is not expected to lead to bisphenol A (BPA) leaching, which has been found in patients’ saliva at higher concentrations after restorative treatments with currently available commercial materials, and could cause adverse health effects, such as allergic reactions and metabolic disorders, even at low concentrations.^40^ Allied with higher conversion, this fact is encouraging in terms of cytotoxicity outcomes, which will be further investigated in future studies. In addition, novel materials can be envisioned based on PG6EMA but using ester-free polymerizable functionalities, to further decrease the potential for degradation by hydrolytic and/or enzymatic mechanisms. Recent studies have demonstrated that the use of acrylamides in dental adhesive formulations increased long-term stability of dental bonds, given the resonance stabilization of the amide bond compared with the ester.^21^ This was true even at pH as low as 1 in buffered aqueous solutions, as well as in the presence of esterases, such as cholinesterase and pseudo-cholinesterase.^21^

In summary, this exploratory study has shown positive features of PG6EMA materials that make them viable alternatives for both adhesive and composite applications. Though the current form still has limitations, strategies have been identified to mitigate these concerns. Future studies will focus on the optimization of adhesive formulations and the evaluation of the interfacial properties derived from their use to bond composite restorations, as well as relevant biological properties, such as cytotoxicity and potential enzymatic degradation.

## Conclusions

In this study, a potential candidate to replace BisGMA in adhesive and composite formulations was identified. Though more studies are needed to optimize the structure of the monomer and composition of the final materials, the reduced stress and higher conversion are promising for composite applications.
